# Dietary plasticity in small Arctic copepods as revealed with prey metabarcoding

**DOI:** 10.1093/plankt/fbae042

**Published:** 2024-09-05

**Authors:** Snorre Flo, Camilla Svensen, Kim Præbel, Bodil Annikki Bluhm, Anna Vader

**Affiliations:** Department of Arctic and Marine Biology, Faculty of Biosciences, Fisheries and Economics, UiT The Arctic University of Norway, Framstredet 39, 9019 Tromsø, Norway; Department of Arctic Biology, The University Centre in Svalbard, PO Box 156, N-9171 Longyearbyen, Svalbard, Norway; Department of Arctic and Marine Biology, Faculty of Biosciences, Fisheries and Economics, UiT The Arctic University of Norway, Framstredet 39, 9019 Tromsø, Norway; Department of Forestry and Wildlife Management, Inland Norway University of Applied Sciences, PO Box 400 Vestad, 2418 Elverum, Norway; Norwegian College of Fishery Science, UiT The Arctic University of Norway, Muninbakken 21, 9037 Tromsø, Norway; Department of Arctic and Marine Biology, Faculty of Biosciences, Fisheries and Economics, UiT The Arctic University of Norway, Framstredet 39, 9019 Tromsø, Norway; Department of Arctic Biology, The University Centre in Svalbard, PO Box 156, N-9171 Longyearbyen, Svalbard, Norway

**Keywords:** predator, prey, trophic interactions, DNA metabarcoding, small copepods, Arctic

## Abstract

**Objectives:**

Small copepods (<2 mm) compose an important constituent of the Arctic marine food web, but their trophic interactions remain largely unexplored, partly due to methodological limitations.

**Methods:**

We here characterize the prey of the abundant cyclopoid *Oithona similis*, harpacticoid *Microsetella norvegica* and calanoid *Microcalanus* spp. from the Arctic Barents Sea and Nansen Basin during four seasons using brute force prey metabarcoding of the 18S rRNA gene.

**Key findings:**

Chaetognaths were unexpectedly the most consistently identified taxa and composed 47% of all prey reads. Some taxa were seasonally important, including diatoms in April–May (43%), dinoflagellates in December (15%) and March (17%), and urochordates in August (20%). Compositional differences among species were also discernible, and the *M. norvegica* diet was significantly different from both *O. similis* and *Microcalanus* spp. The diets varied nevertheless more with season than species despite the inherent trophic traits that distinguish the ambush-predator *O. similis*, chemosensoric particle-chaser *M. norvegica* and current-feeding *Microcalanus* spp.

**Conclusions:**

Our results thus indicate that dietary plasticity is common in small Arctic copepods, regardless of their behaviors or strategies for finding sustenance. We further hypothesize that such plasticity is an important adaptation in systems where prey availability is highly seasonal.

## INTRODUCTION

Copepods dominate the zooplankton of the marine Arctic both in terms of abundance (Auel and Hagen, 2002) and biomass (Hirche and Mumm, 1992; Mumm *et al.*, 1998). The high prevalence of copepods in the Arctic has historically been attributed to large taxa like *Calanus* spp. (Gallienne *et al.*, 2001; Turner, 2004), but the abundance of small copepods (≤2 mm prosome length, Roura *et al.*, 2018) often exceeds that of *Calanus* spp. (Auel and Hagen, 2002; Hirche and Kosobokova, 2011), and occasionally in terms of biomass (Gallienne *et al.*, 2001; Arashkevich *et al.*, 2002; Svensen *et al.*, 2011). Small copepods compose important prey for many larger zooplanktivores such as larval fish (Turner, 1984), carnivorous copepods (Fleddum *et al.*, 2001), chaetognaths (Sullivan, 1980), amphipods (Dischereit *et al.*, 2022) and ctenophores (Stoecker *et al.*, 1987; Purcell *et al.*, 2010), but their own diets remain largely unexplored due to disproportionate focus on larger species, and due to methodological limitations.

Arctic small copepods consist of species whose adult body size is less than 2 mm and includes *Microcalanus pygmaeus*, *Microcalanus pusillus*, *Oithona similis*, *Microsetella norvegica*, *Triconia* (*Oncaea*) *borealis* and *Pseudocalanus* sp. (Auel and Hagen, 2002). They operate in the same epibathypelagic water masses (Kosobokova and Hirche, 2000; Barth-Jensen *et al.*, 2022), and endure nutritionally variable systems where prey are highly diluted (Yen, 2000; Van Duren and Videler, 2003; Tyrell *et al.*, 2020) and larger predators are concomitantly abundant (e.g. chaetognaths, amphipods, carnivorous copepods). They must all balance the trade-off between risk and reward in searching for prey, and have developed distinct modes of prey acquisition, and sophisticated sensory organs to do so (Kiørboe, 2011; Kjellerup and Kiørboe, 2012). To investigate if prey compositions differ among small copepods with different trophic traits, we studied the prey of co-occurring Arctic cyclopoid *O. similis*, harpacticoid *M. norvegica* and calanoid *Microcalanus* spp. (*M. pusillus/M. pygmaeus*) for the first time using prey metabarcoding.

The three investigated species display differences in their trophic biology. *O. similis* (Claus, 1866) is incredibly abundant and has colonized virtually every epipelagic system of the world’s oceans (Paffenhöfer, 1993; Gallienne and Robins, 2001). *Oithona* spp. are active ambush-feeders that invest little in locomotion for prey search (Svensen and Kiørboe, 2000; Saiz *et al.*, 2003). Instead, *O. similis* utilizes mechanosensory to perceive fluid disturbances caused by sinking or swimming prey (Kiørboe and Visser, 1999; Svensen and Kiørboe, 2000), and lunges at prey when within range (Kiørboe, 2011). *Oithona* spp. appear to feed on a wide range of organisms, but may prefer motile over non-motile prey (Uchima and Hirano, 1988; Turner and Granéli, 1992; Atkinson, 1995), especially ciliates (Turner and Granéli, 1992; Castellani *et al.*, 2005; Zamora-Terol *et al.*, 2013; Svensen *et al.*, 2019). They prefer heterotroph over autotrophs protists (Nielsen and Sabatini, 1996; Lonsdale *et al.*, 2000; Granéli and Turner, 2002), although the Antarctic *O. similis* have been found to feed on diatoms (Hopkins and Torres, 1989; Pond and Ward, 2011). Moreover, *Oithona* spp. may utilize particulate organic matter for food (González and Smetacek, 1994; Green and Dagg, 1997; Svensen and Nejstgaard, 2003), but the importance of coprophagous behavior for the Arctic ecotype is disputed (Reigstad *et al.*, 2005).

*M. norvegica* (Boeck, 1865) is broadly distributed in Pacific, Atlantic and sub-Arctic Oceans, and one of few harpacticoid copepods that permanently occupy pelagic waters (Boxshall, 1979). *M. norvegica* is likely a proponent of cruising chemosensory, which involves searching, perceiving and following plumes of dissolved organic molecules that particles and prey leave in their wake (Poulet and Ouellet, 1982; González and Smetacek, 1994; Maar *et al.*, 2006). There exist multiple reports of *M. norvegica* attached to aggregates of marine snow, and especially appendicularian houses (Alldredge, 1972; Ohtsuka *et al.*, 1993; Green and Dagg, 1997; Uye *et al.*, 2002; Maar *et al.*, 2006; Koski *et al.*, 2007). The houses themselves also become colonized by diverse pico-, nano- and microplankton (Lombard *et al.*, 2010), and are therefore potentially rich in other putative prey. However, recent experimental incubations with field-collected autotrophs, detritus and appendicularian houses indicated that *M. norvegica* preferred autotrophs over marine snow (Koski *et al.*, 2020), and algal aggregates over houses (Koski and Lombard, 2022).

The small calanoid *Microcalanus* spp. copepods compose a species-complex with the Arctic *M. pusillus* (Sars G. O., 1903) and the bipolar *M. pygmaeus* (Sars G. O., 1900). While there exist very few studies for *Microcalanus* spp., it is expected that like most calanoid copepods, *Microcalanus* spp. acquire prey through current feeding (Kiørboe, 2011). Whether *Microcalanus* spp. are capable of chemosensory or hydromechanical sensing is unknown, but the former may seem probable with chemoreceptors being identified in other calanoids (Friedman and Strickler, 1975). The prey of *Microcalanus* spp. has, to the best of our knowledge, only been studied in the Antarctic *M. pygmaeus* (Hopkins, 1985, 1987; Hopkins and Torres, 1989), which according to gut inspections, may have a preference for phytoplankton, particularly diatoms, which constituted more than 80% of the identified prey (Hopkins and Torres, 1989).

The main goal of this study was to investigate the prey of *O. similis*, *M. norvegica* and *Microcalanus* spp. from the Barents Sea and Arctic Ocean. By collecting three species and environmental parameters at three locations during four distinct seasons, we aimed to holistically characterize both prey and the drivers behind its variation. We expected a large part of the variation to be explained by seasonality due to the extreme environmental shifts that shape their ambient communities and thus prey availability in the Arctic (Marquardt *et al.*, 2016). Specifically, we expected primary producers to be the dominant prey during the productive period, while small hetero- and mixotrophs would dominate in non-productive periods. Spatial differences in prey were likewise expected, since the Atlantic Barents Sea shelf (Shelf S and Shelf N) and Arctic Nansen Basin (Basin) receive different water masses and prey through advection (Falk-Petersen *et al.*, 2015; Wassmann *et al.*, 2015). We hypothesized that the three species—though overlapping in size and distribution—would find different prey due to their distinctive feeding modes. Thus, we expected *O. similis* to find active and/or motile heterotrophs like ciliates, dinoflagellates or juvenile metazoans, but also sinking particles and aggregates heavy enough to cause fluid disturbances. *M. norvegica* would likely feed on diatoms (Koski and Lombard, 2022), sinking decomposing aggregates (appendicularian houses, fecal pellets) and potentially on their associated communities of colonizers. *Microcalanus* spp. was expected to feed on diatoms (Hopkins, 1987), although particles or other auto- and heterotroph protists, particularly those without innate locomotion may be captured in its feeding currents. Lastly, we specifically aimed to use prey metabarcoding to uncover unknown interactions, which—in line with recent years of trophic studies, has spurred the discovery of new avenues for scientific research.

## MATERIALS AND METHODS

**Fig. 1:**
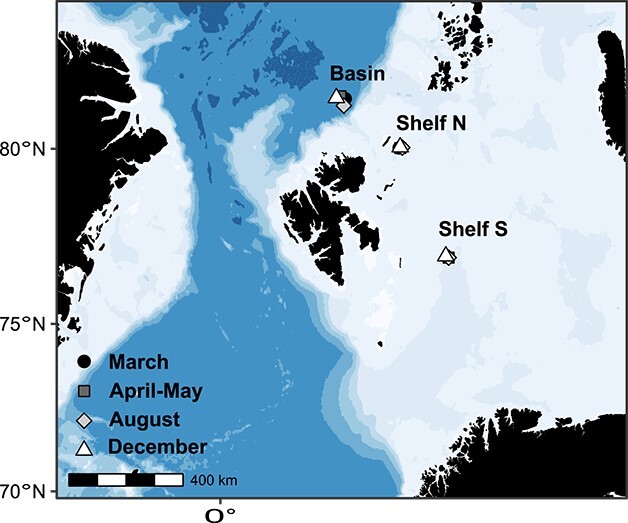
Map of stations located in the Barents Sea (Shelf S and Shelf N) and Nansen Basin (Basin). All stations were sampled on all four seasonal cruises (shapes and colors).

Copepod collection: Mesozooplankton were collected in vertical 64-μm Bongo-net (60 cm, Hydro-Bios) hauls (ascent 0.3 ms^−1^, descent 0.5 ms^−1^) from different seasons and stations in the central and northern Barents Sea (Shelf South, Shelf North) and Nansen Basin (Basin) as indicated in Fig. 1 and Table I. Net samples were processed immediately by removing all large and/or gelatinous animals and sieved (64 μm) to discard seawater. Ice-cold ethanol (96%, −20°C) was then used to rinse the mesozooplankters, and to transfer them to a sample container. The container was topped up with ice-cold ethanol and stored at −20°C.

**Table I:** Overview of copepods collected from four seasons and three stations and the positions of those (Lat/Lon)

Season	Station	Lat (°N)	Lon (°E)	Date	Sd (m)	Bd (m)	Sea-ice	Chl *a* max	Os	Mp	Mn
Mar	Shelf S	75.9999	31.2196	05.03.21	300	324	No	0.010	14	14	14
	Shelf N	79.7662	33.8264	09.03.21	320	340	Yes	0.014	14	14	7
	Basin	81.9989	29.8381	18.03.21	1 000	3 334	Yes	0.021	14	14	NA
Apr-May	Shelf S	76.0000	31.2202	30.04.21	300	326	No	1.664	14	13	13
	Shelf N	79.7438	33.9800	04.05.21	320	337	Yes	2.127	14	14	6
	Basin	82.1422	29.1633	13.05.21	1 000	3 494	Yes	0.288	14	14	NA
Aug	Shelf S	76.0000	31.2200	08.08.19	300	321	No	1.218	14	14	14
	Shelf N	79.7211	34.3182	12.08.19	330	341	No	1.366	14	14	14
	Basin	81.8291	28.8017	21.08.19	1 000	2 993	Yes	1.737	14	13	7
Dec	Shelf S	76.0870	31.0010	13.12.19	300	333	No	0.040	14	14	14
	Shelf N	79.7700	34.0520	08.12.19	300	326	Yes	0.017	14	14	14
	Basin	82.1610	28.1540	04.12.19	1 000	3 660	Yes	0.044	14	14	NA

The maximum sampling depth (Sd; m), bottom depth (Bd; m), whether sea-ice was present (Yes/No) and the recorded maximum total chlorophyll *a* (Chl *a* max; mg m^−3^) are presented for each net-sampling event. The numbers of biological replicates of each copepod picked and whose diet was assessed are shown with species acronyms (*O. similis*; Os, *Microcalanus* spp.; Mp, *M. norvegica*; Mn). Stations sampled correspond to the Nansen Legacy stations P1 (Shelf S), P4 (Shelf N) and P7 (Basin). Seasons sampled correspond to the Nansen Legacy seasonal cruises Q1 (March), Q2 (April–May), Q3 (August) and Q4 (December).

Initial sorting and DNA extraction: Up to 14 biological replicates of *M. norvegica*, *Microcalanus* spp. and *O. similis* were identified and picked per station and cruise under a stereomicroscope (Table I). All copepods were individually and thoroughly rinsed three times in Milli-Q water (MQ), transferred to tissue lysis (TL) buffer (E.Z.N.A Tissue DNA kit, Omega Bio-tek) and stored at −20°C. DNA was extracted per manufacturer’s protocol (Tissue Spin Protocol, E.Z.N.A Tissue DNA kit, Omega Bio-Tek), but with 2 × 50 μL elution buffer, and by incubating on a tabletop thermocycler (300 rpm, 70°C, 3 hours). One extraction negative was included with each batch of samples and was processed identically to real samples.

Molecular preparations, sequencing and bioinformatics. Initial testing of DNA extracts, metabarcode PCR, library preparation, sequencing and bioinformatical processing was achieved as outlined in Flo *et al.* (2024). We therefore only summarize the key methodological choices made here. For sequencing, we used a one-step PCR protocol to target a short hypervariable fragment of the 18S SSU rRNA V7 region (~100–110 bp) with 18S_allshorts primers (Forward 5′-TTTGTCTGSTTAATTSCG- 3′, and Reverse 5′-GCAATAACAGGTCTGTG-3′) (Guardiola *et al.*, 2015). Amplicons from 456 samples including extraction negatives were sequenced on a NovaSeq6000 platform using 150 bp paired-end chemistry (Novogene, China). A total of ~5.4 billion paired-end raw reads were obtained, and further processed using custom scripts (available online: https://github.com/snflo/bruteforce) based on OBITools (v. 1.2.12, Boyer *et al.*, 2016) and VSEARCH (v. 2.9.1, Rognes *et al.*, 2016) software suites and the unoise3 algorithm (Edgar, 2016). Resulting zero-radius Operational Taxonomic Units (zOTUs) were assigned to taxonomy of the Protist Ribosomal database (PR2, v.4.14.0, Guillou *et al.*, 2013) using blastn (BLAST+, v. 2.8.1, Camacho *et al.*, 2009).

Filtering: To obtain a dataset with putative prey only, the assigned reads were subjected to a two-step filtration process in R studio (v. 4.1.3). Firstly, the reads were manually curated based on taxonomy using functions in the “tidyverse” suite (Wickham *et al.*, 2019). All reads assigned to maxillopoda were discarded. We acknowledge that other copepods may compose a food source, but the short read length necessitated by the approach did not allow for distinguishing the DNA from copepod prey and host. Nor did the fragment allow for distinction of *M. pusillus* and *M. pygmaeus*, and we therefore operate with *Microcalanus* spp. for the remainder of this work. Taxa known to interact with copepods (any Copepoda) in symbiosis (parasitism, commensalism and mutualism) were recorded from current literature (Cleary and Durbin, 2016; Cleary *et al.*, 2017; Bass *et al.*, 2021), and discarded from the dataset. Identified symbionts mainly consisted of parasitic protist taxa (e.g. Apicomplexa, Syndiniales, Blastodiniaceae and Apostomia). Group-IV *Hematodinium* (Syndiniales) zOTUs were also discarded due to confirmed parasitic interactions with different crustaceans (Stentiford and Shields, 2005; Zamora-Terol *et al.*, 2021). We identified and discarded several zOTUs of terrestrial seed-plants (Embryophyta), insects and mammals (e.g. *Homo sapiens*), and putative contaminants from the marine environment, notably gelatinous organisms (Cnidaria, Cthenophora). We acknowledge that gelatinous taxa may have dietary importance, but we consider it equally or more plausible that they came from the ambient volume of the fixed sample which the copepods were picked from. A final decontamination step aimed at identifying any remaining and/or cryptic contaminants was achieved using the prevalence method implemented in Decontam (Davis *et al.*, 2018). The dataset after this step is referred to as the putative prey and included numerical abundances (counts) of 22 610 sequence variants (52 165 786 sequence reads), their taxonomy and distribution among 437 copepods.

Environmental data: We obtained various environmental datasets from the same cruises and stations to explore copepod diets in relation to their *in situ* environments. To assign water-column depths to the copepod predators, and to connect them to other parameters at the depths they were sampled, we obtained quantitative data on zooplankton vertical distribution from the same stations. These data were generated from samples taken at different depth intervals (0–20, 20–50, 50–100, 100–200, etc.) using multinet Midi (HydroBios, 64 μm). The dataset holds quantities of all identified zooplankton including the copepods of the current paper, and with resolution of different life stages (Wold *et al.*, 2023). First, we found the vertical distribution intervals containing at least 90% of adult and copepodite V life stages. All 90% intervals were calculated separately for all cruise-station-species combinations. The three copepods may thus have different depth intervals although being collected from the same bongo-net haul (i.e. the 90% depth intervals of *O. similis* and *Microcalanus* spp. from August at Shelf S are 0–50 and 100–325 m, respectively). We then used the minimum and maximum depths to calculate values of environmental parameters as the means of values recorded within the intervals. This way we related the prey profiles of individual copepods to an extended set of mean values of potentially relevant parameters. The parameters included (i) pigment-data (Chlorophyll *a*; total (Chl *a*, μg L^−1^) and 10 μm fraction (Chl *a_10_*, μg L^−1^), Phaeopigment; total (Phaeo, μg L^−1^) and 10 μm fraction (Phaeo_10_, μg L^−1^) (Vader *et al.*, 2021), (ii) particulate organic matter (carbon; POC, mg cm^−3^, nitrogen; PON, mg cm^−3^, carbon:nitrogen ratios; CN, mol:mol (Marquardt, 2022c, 2022d, 2022a, 2022b), (iii) CTD and additional sensory data including temperature (T, °C), salinity (S, PSU), dissolved oxygen (ml L^−1^), Chl *a* fluorescence converted to Chlorophyll *a* concentration (μg L^−1^), photosynthetically available radiation (PAR, mol photons m^−2^ s^−1^) and colored dissolved organic matter (cDOM, μg L^−1^) (Gerland, 2022; Ludvigsen, 2022; Reigstad, 2022; Søreide, 2022). In addition, we obtained a single numerical depth parameter by calculating the mean depth of each copepod species at each station.

Pelagic prey field profiling from water sample metabarcoding. Pelagic water samples were collected at all stations from four depths (10 m, deep Chl *a* maximum, 200 m and bottom—10 m) with a Niskin rosette. Sample material was collected on 0.22 μm Sterivex filters (Merck, Darmstadt, Germany) with a peristaltic pump (Masterflex), frozen immediately and stored at −80°C until extraction. DNA was extracted using the DNA Power Water Sterivex kit (QIAGEN, Hilden, Germany) according to manufacturer’s protocol for vacuum manifold extraction. The V4 region of the 18S rRNA gene was amplified by PCR with the eukaryotic universal primers V4F_illumina (5’-CCAGCASCYGCGGTAATTCC–3′) and V4R_AZig_illumina (5'-ACTTTCGTTCTTGATYRATGA–3′) (Piredda *et al.*, 2017). PCR for sequencing was carried out with an initial denaturation step (30 s, 98°C), and 30 cycles of denaturation (10 s, 98°C), annealing (30 s, 55°C) and elongation (30 s, 72°C) and a final elongation (4:30 min, 72°C). The libraries were prepared and sequenced using Illumina MiSeq technology with 2 x 300 bp paired-end chemistry at the Integrated Microbiome Resource (IMR, Canada). The reads included in water community profiles were bioinformatically treated similarly to prey reads, and thus include the putative prey identifiable by the method (i.e. not putative symbionts or contaminants that were otherwise abundant, like Syndiniales or Ctenophora).

**Fig. 2:**
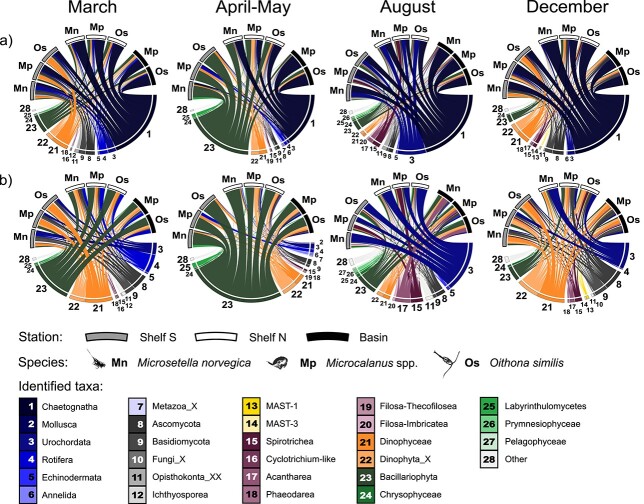
(**a**)Relative composition of prey of copepods *M. norvegica*, *Microcalanus* spp. and *O. similis* and during four seasons (March, April–May, August, December), and at three stations in the northern Barents Sea and adjacent Nansen basin (Shelf S; gray ribbon, Shelf N; white ribbon, Basin; black ribbon). The widths of the interactions between prey taxa (bottom, numbered) and copepods (top, acronyms) are proportional to prey RRA. Only the 15 most abundant prey classes (based on RRA) for each season were plotted. All copepod ribbons (Mn for *M. norvegica*, Mp for *Microcalanus* spp. and Os for *O. similis*) are the averaged prey compositions from up to 14 replicates. In (**b**) Chaetognatha have been removed to provide a clearer representation of other prey classes.

**Fig. 3:**
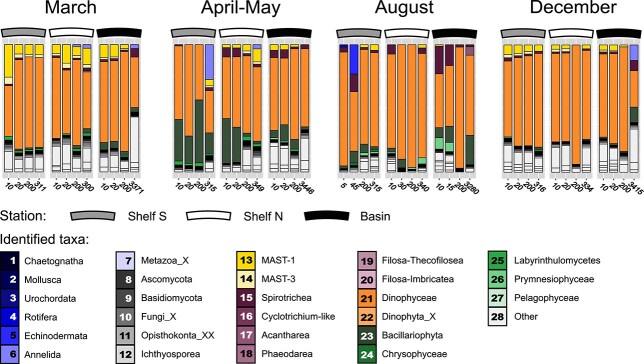
Relative composition of pelagic community taxa during four seasons (March, April–May, August, December) and at three stations in the northern Barents Sea and adjacent Nansen basin (Shelf S; gray ribbon, Shelf N; white ribbon, Basin; black ribbon). The horizontal axis ticks denote the four standard depths (10 m, deep Chl *a* maximum depth, 200 m and bottom 10 m) at which the pelagic communities were assessed by water sampling. Pelagic biota are colored identically to prey found in copepod gut samples.

Exploratory data analyses: We used a combination of tidyverse and phyloseq packages (1.36.0, McMurdie and Holmes, 2013) to wrangle and store data in R, primarily vegan (2.6.2, Oksanen *et al.*, 2019) to perform ecological analyses, and ggplot2 (3.3.6, Wickham, 2016) to visualize findings. First, numerical count data was normalized to comparable values by transforming counts to relative read abundances (RRA, %). To limit the complexity of figures, we chose to present prey RRA agglomerated at class-level taxonomy (Fig. 2a and b). The same agglomerated RRA data were used to calculate mean prey compositions and standard errors of the mean across different sampling groups (seasons, stations, species, Table II). Stacked bar plots visualizing the composition of pelagic prey field communities were made using RRA (Fig. 3), but only taxa that both accounted for more than 1% of the sample-wise RRA and were represented in the top 15 prey taxa in copepod diets were colorized.

**Table II:** Average RRA (%) ± standard error of the mean of copepod prey in all samples, and according to season, stations and species sampled

**PR2 division**	**PR2 class**	**All** **(*n* = 437)**	**March** **(*n* = 105)**	**April–May** **(*n* = 102)**	**August** **(*n* = 118)**	**December** **(*n* = 112)**	**Shelf S** **(*n* = 166)**	**Shelf N** **(*n* = 153)**	**Basin** **(*n* = 118)**	**Mn** **(*n* = 103)**	**Mp** **(*n* = 166)**	**Os** **(*n* = 168)**
Ciliophora	Cyclotrichium_like	0.1±0.1	0.3±0.3		0.1±0.0		0.2±0.2	0.1±0.0		0.4±0.3		
	**Spirotrichea**	**2.2±0.5**	0.1±0.0	0.6±0.3	**5.5±1.3**	**2.4±1.0**	**2.3±0.7**	**3.4±1.0**	0.6±0.3	0.4±0.1	**3.6±0.9**	**2.0±0.7**
Dinoflagellata	**Dinophyceae**	**6.6±0.7**	**11.5±1.9**	**5.3±1.4**	**1.6±0.3**	**8.4±1.5**	**11.6±1.7**	**3.8±0.7**	**3.0±0.3**	**5.1±1.4**	**5.7±0.9**	**8.3±1.4**
	**Dinophyta_X**	**4.5±0.6**	**4.9±1.1**	**5.1±1.0**	**1.7±0.8**	**6.4±1.4**	**3.9±0.7**	**3.9±0.7**	**6.1±1.6**	**1.6±0.3**	**5.6±1.0**	**5.1±1.0**
Breviatea	Breviatea_X					0.2±0.2	0.1±0.1			0.2±0.2		
Lobosa	Tubulinea									0.1±0.1		
Chlorophyta	Pyramimonadophyceae				0.1±0.0	0.1±0.0		0.1±0.0	0.1±0.0		0.1±0.0	
Cryptophyta	Cryptophyceae					0.1±0.1				0.1±0.1		
Haptophyta	Prymnesiophyceae	0.3±0.1		0.1±0.1	0.9±0.2	0.1±0.0	0.3±0.1	0.4±0.1	0.1±0.0	0.5±0.2	0.2±0.1	0.3±0.1
Picozoa	Picozoa_X				0.1±0.1				0.1±0.1	0.1±0.1		
Choanoflagellida	Choanoflagellatea	0.1±0.0	0.1±0.1			0.1±0.1	0.1±0.1		0.1±0.1			0.1±0.1
Fungi	**Ascomycota**	**4.4±0.5**	**5.9±1.3**	**1.7±0.3**	**2.9±0.7**	**7.0±1.4**	**5.2±1.1**	**3.9±0.7**	**3.8±0.8**	**4.5±1.3**	**5.9±0.9**	**2.8±0.6**
	**Basidiomycota**	**2.7±0.5**	**4.3±1.2**	0.7±0.2	**1.0±0.2**	**5.0±1.4**	**4.0±1.0**	**1.1±0.2**	**3.1±1.0**	**4.3±1.5**	**2.1±0.5**	**2.5±0.7**
	Fungi_X					0.1±0.1		0.1±0.0				
	Mucoromycota		0.1±0.1									
Mesomycetozoa	Ichthyosporea	0.1±0.1	0.2±0.2						0.2±0.2		0.1±0.1	
Metazoa	Annelida	0.3±0.1		0.9±0.5		0.2±0.1	0.1±0.1	0.5±0.3	0.2±0.1	0.1±0.1	0.5±0.3	0.1±0.0
	Arthropoda					0.1±0.1			0.1±0.1			
	**Chaetognatha**	**47.2±1.4**	**45.8±2.9**	**35.4±3.1**	**49.7±2.4**	**56.6±2.8**	**31.1±2.1**	**52.2±2.2**	**63.8±2.4**	**46.2±3.2**	**47.3±2.3**	**47.8±2.3**
	Echinodermata	0.4±0.1	0.6±0.5	0.1±0.0	0.6±0.2	0.1±0.0	0.3±0.1	0.1±0.0	0.8±0.5	0.3±0.1	0.6±0.4	0.2±0.0
	Metazoa_X	0.1±0.0	0.1±0.0	0.1±0.0	0.1±0.0			0.1±0.0	0.1±0.0	0.1±0.0	0.1±0.0	0.1±0.0
	Mollusca					0.1±0.0	0.1±0.0			0.1±0.0		
	Nematoda	0.1±0.0				0.2±0.1		0.1±0.1			0.1±0.1	
	Nemertea	0.1±0.0			0.2±0.2		0.1±0.1				0.1±0.1	
	**Rotifera**	**1.8±0.3**	**5.3±1.0**	**1.9±1.0**	0.1±0.0	0.1±0.0	**1.9±0.8**	**1.9±0.4**	**1.5±0.3**	**1.5±0.9**	**2.5±0.7**	**1.3±0.2**
	**Urochordata**	**8.1±0.7**	**5.4±1.5**	**1.5±0.3**	**19.6±1.9**	**4.6±0.7**	**10.1±1.3**	**11.1±1.4**	**1.5±0.2**	**13.1±2.1**	**6.0±0.8**	**7.2±1.1**
Opisthokonta_X	**Opisthokonta_XX**	**1.3±0.3**	**2.0±1.1**	0.1±0.0	**2.8±0.8**	0.3±0.1	**2.9±0.9**	0.5±0.1	0.2±0.1	**1.8±1.0**	**1.4±0.6**	**1.0±0.4**
Cercozoa	Endomyxa-Ascetosporea				0.1±0.1				0.1±0.1			0.1±0.1
	Filosa-Imbricatea	0.2±0.1			0.7±0.4	0.1±0.0	0.4±0.3	0.2±0.1		0.7±0.4	0.1±0.0	0.1±0.0
	Filosa-Thecofilosea	0.1±0.1		0.3±0.2	0.2±0.1		0.2±0.2	0.1±0.0			0.2±0.2	0.1±0.0
	Phaeodarea	0.2±0.1	0.3±0.1	0.1±0.1		0.3±0.3	0.1±0.0		0.5±0.3	0.1±0.0	0.2±0.1	0.2±0.2
Radiolaria	**Acantharea**	**1.1±0.2**		0.1±0.0	**2.8±0.6**	**1.1±0.3**	0.8±0.2	0.4±0.1	**2.4±0.6**	**1.0±0.4**	**1.2±0.4**	0.9±0.2
	Polycystinea		0.1±0.1						0.1±0.1		0.1±0.1	
Ochrophyta	**Bacillariophyta**	**15.9±1.2**	**12.2±1.3**	**42.8±3.2**	**6.5±1.3**	**4.8±1.2**	**21.4±2.5**	**14.2±1.5**	**10.4±1.4**	**14.8±2.6**	**14.4±1.7**	**18.1±2.0**
	**Chrysophyceae**	0.5±0.1	0.2±0.1	0.4±0.2	**1.0±0.3**	0.4±0.1	0.5±0.1	0.5±0.2	0.5±0.3	0.3±0.2	0.5±0.2	0.6±0.2
	Dictyochophyceae	0.1±0.1			0.3±0.3			0.2±0.2			0.2±0.2	
	Pelagophyceae	0.1±0.0			0.2±0.1	0.1±0.1	0.2±0.1	0.1±0.0		0.1±0.1		0.1±0.1
	Xanthophyceae				0.1±0.1							
Opalozoa	MAST-3	0.1±0.1				0.4±0.3	0.3±0.2			0.4±0.4		0.1±0.0
Pseudofungi	MAST-1	0.1±0.0	0.1±0.0	0.1±0.0	0.1±0.1	0.3±0.1	0.1±0.1	0.2±0.1	0.1±0.1	0.2±0.1	0.1±0.0	0.1±0.1
Sagenista	**Labyrinthulomycetes**	0.9±0.2	0.4±0.1	**2.4±0.8**	0.6±0.3	0.2±0.1	**1.5±0.5**	0.7±0.1	0.3±0.0	**1.9±0.9**	0.5±0.1	0.7±0.1
Stramenopiles_X	Stramenopiles_XX				0.1±0.0				0.1±0.0			

Parentheses denote number of in-group samples used to calculate average RRA. Average values smaller than 0.1% were removed from the table, and values greater than 1% are shown in bold. Copepod consumers are abbreviated Os for *O. similis*, Mp for *Microcalanus* spp. and Mn for *M. norvegica*.

Canonical correspondence analyses: Canonical correspondence analyses (CCAs) were conducted with the full set of prey samples (*N* = 437) and a limited set of environmental constraints. The samples were prey compositions in the form of RRA agglomerated to class-level taxonomy (with a total of 83 classes). Prior analysis, we evaluated the parameters to include seemingly uncorrelated parameters only with the pairs function. The retained parameters included Chlorophyll *a* (Chl *a*, μg L^−1^), particulate organic carbon (POC, mg m^−3^), carbon:nitrogen ratio (CN, mol:mol), mean depth of copepods (depth, m), salinity (S, psu), temperature (T, °C), photosynthetically available radiation (PAR, mol photons m^−2^ s^−1^), species, latitude (Lat, deg. N), longitude (Lon, deg. E) and bottom depth (Bd, m). The full dataset was evaluated first, beginning with testing transformation of RRA and a full model with all twelve constraints. The best full model was found using fourth root transformed RRA values (^0.25). Then, model constraints were evaluated stepwise by backwards selection, and for each new model ANOVA-like permutation tests for CCA (*anova.cca*) were used to find non-significant terms (*P* > 0.001), which were subsequently discarded. This process was repeated for every new model iteration, until a model with only significant terms (*P* ≤ 0.001) was left. For readability, all overlayed constraints and accompanying arrows were scaled (*ordiArrowMul*, fill = 0.8).

To investigate if the prey profiles would cluster by predator or stations when seasonality was accounted for, CCAs were performed on subsets from each season separately. The resulting four models were generated as before, but scaling of overlayed constrains was moderated separately for each plot (fill = 0.80, 0.85, 0.84 and 0.85, for March, April–May, August and December in Fig. 5, respectively).

Permutational Multivariate Analysis of Variance: We used Permutational Multivariate Analysis of Variance (PERMANOVA, *adonis2*) to test for differences in diets with season, station or species as grouping variables (Oksanen *et al.*, 2019). Between-group homogeneity of dispersion was assessed with *betadisper*. Pairwise PERMANOVAs (*pairwise.adonis2*, Martinez, 2020) were used to compare and identify the levels in groups in which the diets differed significantly. *P*-values of pairwise tests were adjusted with the false discovery rate method of Benjamini–Hochberg (Benjamini and Hochberg, 1995). Additional tests were performed on the subsets of the data (March, April–May, August and December) to identify differences in diet based on station or species without the strong influence of season.

SIMPER and BLAST: We used Similarity Percentages analyses (SIMPER, *simper*, Clarke, 1993) to identify the prey taxa contributing most to differences in composition. We ran the analyses with permutations (*n* = 10 000) in both ungrouped and grouped tests (pairwise comparisons of species, stations and seasons), using Bray–Curtis dissimilarities based on the 5000 most abundant zOTUs overall (based on RRA). The twenty top zOTUs, which at minimum contributed 65% to observed dissimilarity—were compiled for each comparison, and sequences were further assessed by a BLAST to NCBI’s nucleotide archive (nt). Hits were recorded based on their percentage similarity, keeping only the highest scoring. In the case of multiple equally similar hits with varying taxonomies, we chose to summarize the hits to their closest common ancestor.

## RESULTS

Prey read recovery: Sequencing of eukaryote ribosomal DNA produced 5 436 416 402 raw reads from 437 copepod samples and 19 extraction negatives. Of the raw reads, 4 268 371 437 reads in 129 940 zOTUs passed the quality thresholds of our protocol and were subsequently taxonomically assigned. Almost 98% of the assigned reads mapped to Maxillopoda, but this was expected due to the use of a brute-force metabarcoding approach (Flo *et al.*, 2024). Nonetheless, putative prey amounted to 52 million reads in 22 391 zOTUs, making up 1.2% of the assigned reads.

Overall composition of copepod prey and the pelagic prey field community. From metabarcoding of *O. similis*, *Microcalanus* spp. and *M. norvegica* copepods, we identified a range of eukaryote prey including metazoans, fungi, ciliates, radiolarians, dinoflagellates and diatoms (Fig. 2 and Table II). If looking at the global average prey RRA (*n* = 437), metazoan sequence reads dominated over unicellular reads (Table II). The most abundant prey taxa overall were Chaetognatha (47% RRA), Bacillariophyta (16%), Urochordata (8%), Dinophyceae (7%), Dinophyta_X (5%), Ascomycota (4%), Basidiomycota (3%) and Spirotrichea (2%, Table II). In the prey field community (Fig. 3), RRA of unicellular taxa dominated over metazoan taxa. Chaetognath reads were not prevalent, and never surpassed the 1% RRA threshold we used to limit complexity in bar plots (Fig. 3). Instead, Dinophyceae were the most abundant taxa and accounted for between 29 and 96% of the RRA. The other important dinoflagellate prey taxon (Dinophyta_X, Fig. 2a and b) was below 1% RRA in all water samples. Bacillariophytes were mainly abundant in water sampled in April–May at stations Shelf S (17–49% at all depths) and Shelf N (28–35% at 10 and 20 m depths). Deep water samples from the Nansen Basin in August (3 280 m) and December (3 415 m) contained 23 and 12% bacillariophytes, respectively. Marine Stramenopiles group 1 and 3 (MAST-1 and MAST-3) were present year-round, but noticeably more abundant in March and December pelagic communities (Fig. 3).

**Fig. 4:**
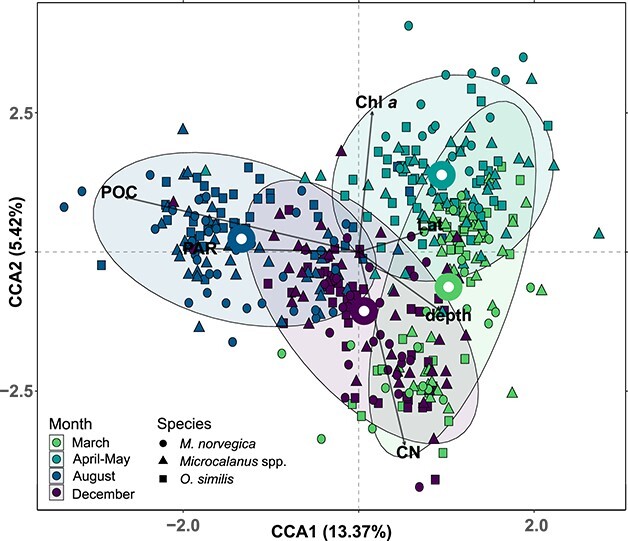
Canonical correspondence analysis of class level prey composition of copepods in all samples. Donut shaped points are the centroids of seasonal clusters. Clusters are encircled by 95% confidence ellipses. The fractions of the total inertia explained (in %) are reported for each constraining axis. Only constraints that were found to be significant by the ANOVA-like permutation tests for CCA (*P* < 0.005) and survived backwards selection are reported.

Seasonal differences in prey composition: To discern the prey characteristics as a function of spatiotemporal scales or consumer, we also summarized the prey compositions across the seasons, stations and species (Table II). The constrained ordination, with its apparent seasonal clustering and parameters (Chl *a*, POC, PAR) as important structuring constraints (Fig. 4)—indicated that seasonality was important in explaining prey compositions. We further tested the importance of seasonality by PERMANOVA, and found that prey composition differed significantly according to the season sampled (*F* = 32.14, *P* < 0.001, homogenous dispersion, Table III). Additional pairwise comparisons of seasonal prey composition were all significant (PERMANOVAs, adjusted *P* < 0.001, Table III). In winter, the prey composition was composed of more dinoflagellates (Dinophyceae and Dinophyta_X combined) constituting 15 and 16% of the diet in December and March, respectively. Fungal sequences from Ascomycota and Basidiomycota were also abundant in winter, contributing a combined 12 and 10% to the diet in December and March, respectively. These “winter seasons” are relatively similar in composition (Fig. 2a), but a few distinguishing features exist, namely the increased abundances of Rotifera (5%) and Bacillariophyta (12%) in March, and that Spirotrichea were abundant in December (2%). Prey composition in April–May was characterized by an increased relative abundance of diatoms (Bacillariophyta; 43%), dinoflagellates (Dinophyceae; 12%, Dinophyta_X; 5%) and Labyrinthulomycetes (2%). The prey composition in August differed from the other seasons by a high RRA of Urochordata (20%), Spirotrichea (6%) and Acantharea (3%) irrespective of target copepod species.

**Table III:** Results of PERMANOVA analyses used to test which parameters were significant predictors of prey composition for all samples (full dataset) and for seasonal subsets of the data (March, April–May, August and December)

Predictor	Full dataset	March	April–May	August	December
**a)** Season Four levels: March, April–May, August, December	PERMANOVA^*^^*^^*^ (*F* = 32.14, *P* < 0.001) Betadisper non-sig Pairwise sig. tests (all ^*^^*^^*^, *P* < 0.001) March vs. April–May March vs. August March vs. December April–May vs. August April–May vs. December August vs. December				
**b)** Station Three levels: Shelf S, Shelf N, Basin	PERMANOVA^*^^*^^*^ (*F* = 22.59, *P* < 0.001) Betadisper^*^^*^^*^ (*P* < 0.001) Pairwise sig. tests Shelf S vs. Shelf N (^*^^*^^*^, *P* < 0.001) Shelf S vs. Basin (^*^^*^^*^, *P* < 0.001) Shelf N vs. Basin (^*^^*^^*^, *P* < 0.001)	PERMANOVA^*^^*^^*^ (*F* = 13.23, *P* < 0.001) Betadisper^*^^*^^*^ (*P* < 0.001) Pairwise sig. tests Shelf S vs. Shelf N. (^*^^*^, *P* < 0.01) Shelf S vs. Basin (^*^^*^, *P* < 0.01)	PERMANOVA^*^^*^^*^ (*F* = 44.46, *P* < 0.001) Betadisper non-sig Pairwise sig. tests Shelf S vs. Shelf N (^*^^*^^*^, *P* < 0.001) Shelf S vs. Basin (^*^^*^^*^, *P* < 0.001) Shelf N vs. Basin (^*^^*^^*^, *P* < 0.001)	PERMANOVA^*^^*^^*^ (*F* = 14.99, *P* < 0.001) Betadisper^*^ (*P* < 0.05) Pairwise sig. tests Shelf S vs. Shelf N (^*^, *P* < 0.05) Shelf S vs. Basin (^*^^*^, *P* < 0.01) Shelf N vs. Basin (^*^^*^, *P* < 0.01)	PERMANOVA^*^^*^^*^ (*F* = 4.78, *P* < 0.001) Betadisper^*^ (*P* < 0.05) Pairwise sig. tests Shelf S vs. Shelf N (^*^^*^, *P* < 0.01) Shelf S vs. Basin (^*^, *P* < 0.05) Shelf N vs. Basin (^*^, *P* < 0.05)
**c)** Species Three levels: *M. norvegica* (Mn), *Microcalanus* spp. (Mp), *O. similis* (Os)	PERMANOVA^*^^*^ (*F* = 3.29, *P* < 0.01) Betadisper non-sig Pairwise sig. tests Os vs. Mn (^*^, *P* < 0.05) Mp vs. Mn (^*^^*^, *P* < 0.01)	PERMANOVA^*^ (*F* = 1.95, *p* = 0.05) Betadisper^*^^*^ (*P* < 0.01) Pairwise sig. tests Os vs. Mn (^*^, *P* < 0.05) Mp vs. Mn (^*^^*^, *P* < 0.01)	PERMANOVA (*F* = 1.81, *p* = 0.098) Betadisper non-sig Pairwise sig. tests Os vs. Mn (^*^, *P* < 0.05) Mp vs. Mn (^*^, *P* < 0.05)	PERMANOVA^*^^*^^*^ (*F* = 3.34, *P* < 0.001) Betadisper non-sig Pairwise sig. tests Os vs. Mp (^*^, *P* < 0.05) Mp vs. Mn (^*^, *P* < 0.05)	PERMANOVA^*^^*^ (*F* = 2.54, *P* < 0.01) Betadisper non-sig Pairwise sig. tests Mp vs. Mn (^*^, *P* < 0.05)

*F*-values denote the pseudo-*F* statistic (magnitude) computed by adonis. Asterisks denote significance (^*^^*^^*^: *P* < 0.001, ^*^^*^: *P* < 0.01, ^*^: *P* < 0.05, non-sig: *P* > 0.05), and all *P*-values from pairwise tests were adjusted for multiple testing using the Benjamini-Hochberg method.

**Fig. 5:**
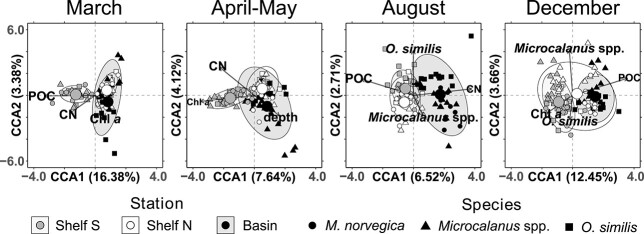
Canonical correspondence analysis of class level prey composition of copepods subset by season. Donut-shaped points are the centroids of station clusters. Clusters are encircled by 95% confidence ellipses. The fractions of the total inertia explained (in %) are reported for each constraining axis. Only constraints that were found to be significant by the ANOVA-like permutation tests for CCA (*P* < 0.005) and survived backwards selection are reported.

Spatial prey differences: Prey composition also differed significantly among stations (PERMANOVA, *F* = 22.59, *P* = 0.001), and all three stations differed significantly from one another (pairwise PERMANOVAs, adj. *P* < 0.001, Table III). However, the sample dispersions within stations were found to be heterogeneous (*P* < 0.001), lowering the confidence in this result. The ellipses (95%) of stations further overlapped to a great extent according to the CCA of all samples, and the centroids were close to one another (Fig. S1). Only when the seasonality was accounted for by sub-setting and ordinating seasonal datasets individually, was it possible to discern that prey compositions clustered according to the station they were collected at (Fig. 5). The strongest regional differences in prey composition were found in April–May (*F* = 44.46, *P* < 0.001, homogenous dispersion), and Shelf S, Shelf N and Basin diets differed significantly from one another (pairwise tests, adj. *P* < 0.001). Diets were less different among stations in December (*F* = 4.78, *P* < 0.001). Some regional differences could nevertheless be identified, such as chaetognath prey being on average more prevalent in the Basin (64%) and at Shelf N (52%) than at the southernmost Shelf S station (31%) (Fig. 2a and Table II).

There were also spatial differences at lower taxonomic levels, with two zOTUs dominating the Chaetognath relative abundances. Using BLAST and NCBI’s nucleotide archive (NT) to verify the taxonomy of taxa that are sparsely covered in the protist ribosomal database, we found that the top two Chateognath zOTUs were identical to *Eukrohnia* spp. (*E. hamata*, *E. bathypelagica*) and *Parasagitta* spp. (*P. elegans*, *P. setosa*) sequences, respectively. The zOTU assigned to *Eukrohnia* spp. reached an average of 44% relative abundance in the basin, as opposed to 7 and 11% at Shelf S and N, respectively. On the contrary, *Parasagitta* spp. was most abundant at shelf stations (Shelf S—19%, Shelf N—36%) and less so in the basin (10%). Bacillariophyta prey were more important at the southern stations Shelf S (21%) and Shelf N (14%) than in the Basin (10%). Urochordata, which was primarily composed of a zOTU with identical sequence to *Oikopleura* spp. (*Oikopleura vanhoeffeni*, *Oikopleura labradoriensis*), were likewise more prevalent prey at Shelf S (10%) and Shelf N (11%) than in the Basin (2%). Dinophyceae were particularly abundant at Shelf S (12%), while members of Dinophyta_X were abundant in the Basin (6%).

Prey differences among copepod species. Although the compositional differences in prey among copepod species were not as strong as those among seasons or stations, the species of copepod was still a relevant predictor of diet (PERMANOVA, *F* = 3.29, *P* < 0.01, Table III). Pairwise comparisons showed that the diet of *M. norvegica* differed from the two other species (adj. *P* < 0.05 and < 0.01), whereas *O. similis* and *Microcalanus* spp. were statistically indifferent (adj. *P* = 0.17). When the prey data was divided to account for seasonality, consumer species remained a significant predictor of prey composition in March (*F* = 1.95, *P* = 0.05), August (*F* = 3.34, *P* < 0.001) and December (*F* = 2.54, *P* < 0.01, Table III), but not in April–May (*F* = 1.81, *P* = 0.098).

The *M. norvegica* diet contained an abundant chaetognath component (46%) at levels comparable to the other two consumers (47–48%, Table II). However, a chaetognath zOTU identified to *Parasagitta* sp. composed a higher fraction of the *M. norvegica* prey reads (29% as opposed to 18% in *Microcalanus* spp., and 23% in *O. similis*), and was responsible for 14% of the dissimilarity between *M. norvegica* and the two other consumers (SIMPER, Table IV). Urochordate prey were more important for *M. norvegica* (13%) than for *O. similis* (7%) and *Microcalanus* spp. (6%, Table II). A single appendicularian zOTU characterized to *Oikopleura* spp. (12% in *M. norvegica*) explained 7% of the dissimilarity between *M. norvegica* and the two other consumers (SIMPER, Table IV). Labyrinthulomycetes (class Labyrinthulea) contributed more to the prey composition of *M. norvegica* (1.9%) than it did for *O. similis* (0.7%) or *Microcalanus* spp. (0.5%, Table II). The zOTU responsible for most of the RRA (1.4% in *M. norvegica*) had 100% sequence similarity to *Oblongichytrium* sp. (family Thraustochytriaceae, Table IV).

**Table IV:** zOTUs contributing significantly to dissimilar prey compositions in Microcalanus spp. (Mp), M. norvegica (Mn) and O. similis (Os)

zOTU	Class	a_b	RAa	RAb	CD	p	Id	Accession
10	Chaetognatha	Mp_Mn	18.2	28.8	14	0.02	*Parasagitta setosa,* *Parasagitta elegans*	KX709930.1 KP857142.1
18	Urochordata	Mp_Mn	5.6	12.2	7	0.01	*Oikopleura vanhoeffeni,* *Oikopleura labradoriensis*	MG661056.1 MK621852.1
167	Labyrinthulomycetes	Mp_Mn	0.3	1.4	1	0.04	*Oblongichytrium* sp.	MK234637.1
10	Chaetognatha	Os_Mn	23.3	28.8	14	0.00	*P. setosa,* *P. elegans*	KX709930.1 KP857142.1
18	Urochordata	Os_Mn	6.8	12.2	7	0.00	*O. vanhoeffeni,* *O. labradoriensis*	MG661056.1 MK621852.1
107	Bacillariophyta	Os_Mn	0.9	1.5	1	0.00	*Fragilariopsis kerguelensis, Fragilariopsis* sp.	LR812489.1 MN824024.1
167	Labyrinthulomycetes	Os_Mn	0.5	1.4	1	0.01	*Oblongichytrium* sp.	MK234637.1
14	Chaetognatha	Os_Mp	18.2	22.3	12	0.02	*Eukrohnia hamata,* *Eukrohnia bathypelagica*	KM519853.1 DQ351886.1
12	Dinophyta_X	Os_Mp	2.5	2.8	2	0.01	Uncultured eukaryote	KJ760297.1
84	Spirotrichea	Os_Mp	1.0	3.0	2	0.00	*Parafavella gigantea*	MH673409.1
170	Dinophyta_X	Os_Mp	1.5	1.3	1	0.03	*Oncaea* sp.	MK370211.1
173	Bacillariophyta	Os_Mp	1.0	0.7	1	0.00	*Porosira* sp.*,* *Porosira glacialis,* *Porosira pseudodenticulata*	MH843681.1 MH843667.1 MG022775.1

zOTUs are presented with class (PR2), consumers compared (a_b), average RRA in consumers (RAa and RAb), zOTU contribution to dissimilarity (CD), permutational significance (p) and identified taxa with 100% sequence similarity (Id).

Spirotrich ciliate prey (Spirotrichea) were more abundant for *Microcalanus* spp. (3.6%) than *O. similis* (2.0%) and *M. norvegica* (0.4%) prey compositions (Table II). One zOTU identified to the spirotrich tintinnid Xystonellidae family (*Xystonella longicauda* in PR2, and *Parafavella gigantea* in NCBIs nt) was a significant contributor to the dissimilarity between *O. similis* and *Microcalanus* spp. (Table IV). Likewise, *Eukrohnia* spp. (*E. hamata*, *E. bathypelagica*) was more abundant in *Microcalanus* spp. (22%) than *O. similis* (18%) and explained 12% of the dissimilarity between the two. *O. similis* showed a higher relative abundance of diatoms (18%) than *Microcalanus* spp. (14%) and *M. norvegica* (15%). Of the diatom zOTUs, only *Porosira* sp. contributed significantly to the dissimilarity between *O. similis* and *Microcalanus* spp. diets.

## DISCUSSION

Prey diversity: We studied the diets of *O. similis*, *Microcalanus* spp. and *M. norvegica* because they are abundant Arctic representatives of different copepod lineages (Cyclopoida, Calanoida, Harpacticoida), and feeding traits (ambush predatory, filter-feeding, aggregate feeding). Although coinhabiting the same habitat (Kosobokova and Hirche, 2000; Barth-Jensen *et al.*, 2022), we hypothesized that their trophic traits would translate into species-specific diets. Our results, however, show that other parameters were more important drivers of prey composition. Seasonality was particularly influential, with prey compositions being explained by time of year sampled, and seasonally shifting parameters (PAR, Chl *a*, POC, CN) being important constraining variables. This is perhaps to be expected, since the diversity and abundance of Arctic communities, and thus prey availability, changes drastically in relation to seasonal parameters like nutrients and light (Wilson *et al.*, 2017; Paulsen *et al.*, 2018). From the sequencing data, we detected over 50 million prey sequence reads that identified to a broad consortium of eukaryotes ranging from large metazoans to small unicellular protists and autotrophs. In general, we saw that diets shifted in composition with high relative abundances of hetero- and mixotrophs in winter (chaetognaths, marine fungi, dinoflagellates), diatoms at the Barents Sea shelves during the spring-bloom (April–May), and diets in August were more influenced by heterotroph metazoans (chaetognaths, urochordates).

Are chaetognath prey DNA remnants from fecalia or juveniles? Although seasonality was important, our results indicate that chaetognaths were important prey for the small copepods year-round, and especially at the northern stations Shelf N and Basin, where they composed 52 and 64% of the prey reads, respectively. We must emphasize, however, that these findings bear with them some uncertainty. Adults of the chaetognaths occurring in the region (*Parasagitta elegans*, *Pseudosagitta maxima*, *Eukrohnia hamata*) are large and thus unlikely targets for ingestion. The chaetognaths are furthermore known consumers of copepods (Falkenhaug, 1991; Grigor *et al.*, 2020), and not vice versa, although some copepod genera like the carnivorous *Pareuchaeta* may be capable. We thus find it likely that the strong signal is due to either ingestion of other chaetognath material types, and/or contamination. The uncertainty of how the chaetognath sequences entered the samples highlights an important limitation to DNA-based trophic studies. Since we do not observe feeding directly, we may not be able to conclusively deduce the type of prey source material, nor the nature of its association with the consumer species. It would arguably be wrong to either discard such sequences or to treat them as “full members” of the prey spectrum, given the size of chaetognaths and their observed abundances. In the current study, we therefore cautiously interpret the chaetognath sequences as putative prey, while exploring its potential sources based on current knowledge. We further argue that research with more direct methods of identifying interactions is required to validate its importance as prey for small Arctic copepods (e.g. cinematography, experimental incubations or applied starvation controls).

Alternative mechanisms of uptake may involve feeding on juvenile stages such as eggs or larvae, fecalia or chaetognath remains (e.g. decaying bodies, parts or egg-sacs). Feeding on juvenile stages may be practically feasible if the co-occurring chaetognaths produce small eggs or larvae that exist unprotected in the water-column. A recent study of *P. elegans* disclosed the diameter of its oocytes to be approximately 0.1 mm (Grigor *et al.*, 2017). *P. elegans’* eggs are also released from the adult at an early reproductive stage, and float towards the surface because of their innate buoyancy (Hagen, 1999), making their juveniles potentially available for predation by small copepods. (Cleary *et al.*, 2017) observed high RRA of chaetognaths in *Calanus glacialis*, and also hypothesized feeding on juvenile stages, especially in winter, when other prey were less abundant. We likewise found high RRA of chaetognaths during the winter-like seasons December (57%) and March (46%), but the relative number of prey reads was at comparable levels in August (50%). At the zOTU-level, *P. elegans* was on average most abundant in December (~35% of relative abundance), but these levels are also comparable to the average composition from August (~27% of relative abundance). Hence, our data suggest that putative juvenile feeding is not a phenomenon limited to winter or seasons of low productivity. Still, a consistently high intake of eggs or juveniles demands a consistent supply, and from what we know on a scarcely researched matter—reproduction in both *P. elegans* and *E. hamata* is likely seasonal, with one or two respective spawning seasons yearly (Grigor *et al.*, 2017). It is therefore unreasonable to expect that juvenile feeding alone has supported the consistently high RRA of chaetognaths in the current study.

### Particle-associated feeding

Fecal pellets: Zooplankton are known regulators of vertical carbon flux through egestion, ingestion and/or fragmentation of fecal pellets and other organic particles (Riser *et al.*, 2007). The high abundances of both chaetognath and urochordate reads may thus indicate utilization of particulate material from fecal pellets, body parts, egg-sacs or housings, respectively. Whilst Arctic chaetognaths may reproduce seasonally, we know they are present year-round in the Svalbard region (Grigor *et al.*, 2015). Their fecal material could thus be available year-round, which could partially explain why chaetognath reads are abundant regardless of season. A study using similar methods with the Arctic *Pseudocalanus* sp. identified a significant portion of prey from much larger euphausiids, and similarly hypothesized that it could have originated from feeding on euphasiid fecal pellets (Cleary *et al.*, 2016). Hence, utilization of particulate matter from larger metazoans may be more important than previously realized. How copepods interact with particles likely depends on several factors including mode of feeding, sensory adaptations, and the size-ratio between copepod and particle. Small filter-feeding calanoids (*Acartia* sp., *Temora longicornis*, *Centropages* sp.) have been found to feed mainly on smaller dispersed food particles (Koski *et al.*, 2017), whereas larger filter-feeding *Calanus* spp. and *Pseudocalanus* spp.—in another study, fed on larger settling aggregates and decreased organic particle flux by over 60% in three Arctic locations (van der Jagt *et al.*, 2020). One would thus expect that *Microcalanus* spp.—as a small, filter-feeding calanoid, would be able to exploit small unicellular organisms and food particles, but not large aggregate particles like appendicularian houses (16 mm long, and 12 mm wide in *O. labradoriensis*, Gorsky *et al.*, 2004) or chaetognath fecal pellets (~1.3 mm long, ~ 0.3 mm wide in epipelagic *Parasagitta eneritica*, Dilling and Alldredge, 1993).

Appendicularian prey. That *M. norvegica* uses larvacean houses in some capacity has been known for quite some time (Ohtsuka *et al.*, 1993; Green and Dagg, 1997). It is however unknown if the copepods ingest house material, which is a complex mixture of mucopolysaccharides, and protein (oikosins, Hosp *et al.*, 2012), feed on its colonizers and aggregated particles, or if they use them as benthic-like substrates for some other non-dietary reasons. The increased relative abundance of urochordate reads (with *Oikopleura* spp. comprising the majority) in *M. norvegica* (13% as opposed to 7% or 6% in *O. similis* and *Microcalanus* spp.), suggests an interaction with appendicularian houses that is not occurring to the same extent with the other two copepods. Feeding experiments with North Sea *M. norvegica*, appendicularian houses and algal aggregates (*Phaeocystis* spp. and diatoms) have shown that algal aggregates led to substantially greater pellet production than appendicularian houses (Koski and Lombard, 2022). Seen in context, our results may thus indicate that *M. norvegica* uses appendicularians for food purposes, but that it is rather the particles or organisms collected by the house, or its colonizers, which it actively feeds on, while the appendicularian prey reads may stem from mucous house material glued to exoskeletons despite rigorous washing. In August, prey compositions of *M. norvegica* and *O. similis* differed significantly from *Microcalanus* spp. (*P* > 0.05), but not from one another. While *Microcalanus* spp. did obtain a lower relative abundance of urochordate prey, the cause of their distinctiveness in August is likely multifaceted, with important contributions from other taxa, such as a pronounced tintinnid prey (family Xystonellidae) in *Microcalanus* spp. Compiled, these results suggest that *M. norvegica* depends the most on appendicularians for food, but it is not alone, as both *O. similis* and *Microcalanus* spp. acquired respectably high RRA of the prey type. This suggests that chemosensory is not a prerequisite for utilizing appendicularians for food purposes. Perhaps the mechanosensory of *O. similis* enables it to detect a heavily sinking house, or that *Microcalanus* spp. may scavenge particulate remains of fragmented appendicularians.

Fungi and Labyrinthulomycetes: We acquired many prey sequences from taxa associated with decomposition and remineralization. Most notable of these were the marine fungi (classes Ascomycota, Basidiomycota) and the enigmatic heterotroph protists in Labyrinthulomycetes (class Labyrinthulea). Marine fungi have previously been detected in the prey compositions of Arctic calanoid copepods including *Pseudocalanus* spp. (Cleary *et al.*, 2016), *C. glacialis* (Cleary *et al.*, 2017) and *Calanus finmarchicus* (Yeh *et al.*, 2020). Fungi were most abundant in the winter seasons of March and December, suggesting that fungal prey became viable in low-productive seasons, and potentially when other preferred prey are rare. The Labyrinthulea, however, which have been associated with a range of ecological roles including marine snow degradation (Bochdansky *et al.*, 2017) and parasitism of autotrophs (Scholz *et al.*, 2016)—were most abundant during spring, at Shelf S and more so in *M. norvegica* than the other copepods. Most of the labyrinthulean zOTUs were assigned to the *Oblongichytrium* genus (family Thraustochytriaceae), which also contributed the bulk of the reads. The *Aplanochytrium* genus (family Aplanochytriidae), which has been identified from gut contents of Pacific *Calanus sinicus* (Hirai *et al.*, 2018) and Indian Ocean mesozooplankton (Damare and Raghukumar, 2010), was not detected although entries of the genus existed in the PR2 database (v. 4.14.0). Since *Oblingichytrium* co-occur with diatoms almost exclusively in the diet of *M. norvegica*, it may be suggested that *Oblongichytrium* colonized sinking aggregates, possibly of diatoms, of which *M. norvegica* are proponents of, but which the other two copepods disfavor for “fresher” food particles during the productive spring season. Nevertheless, these findings are to the best of our knowledge the first reports of *Oblongichytrium* prey in any mesozooplankton and should be investigated further to better understand the complexity of marine food webs.

## CONCLUSIONS

We studied the trophic interactions of Arctic *O. similis*, *M. norvegica* and *Microcalanus* spp. for the first time using prey metabarcoding. We experienced that seasonality of Arctic ecosystems overpowered species-specific dietary preferences. During the productive Arctic spring, prey reads were mainly dominated by diatoms, whereas winter prey mainly belonged to heterotroph (chaetognath, fungi) and mixotroph taxa (dinoflagellates). Shelf copepod diets in late summer had a greater contribution from heterotrophs and suggested greater reliance on particulate matter from appendicularian housings and chaetognaths. Of the latter, we find that fecal pellets are the most probable source, but this interaction requires validation. We further identified zOTUs that explained the dissimilarities between consumers, including a novel *Oblongichytrium* prey interaction for *M. norvegica*. In general, DNA metabarcoding proves its usefulness for identifying trophic exchange in marine food webs, and in particular for generating new hypotheses, but the nature of potential interactions remains difficult to describe or verify with DNA metabarcoding alone. Thus, a deepened understanding of the role of small copepods in the Arctic may benefit from studies that verify ingestion, for instance by experimental incubations, starvation controls or through cinematographic studies of prey capture.

## Supplementary Material

Flo_et_al_JPR_Supporting_information_fdae042

## Data Availability

Raw sequence reads and accompanying metadata are available in the NIRD Research Data Archive (https://doi.org/10.11582/2023.00065). All bioinformatic code used to process raw sequences to a zOTU-table is available on github (https://github.com/snflo/bruteforce). The method used is explained in more detail in (Flo *et al.*, 2024).
