# Supplementary material for: Dietary plasticity in small Arctic copepods as revealed with prey metabarcoding
Source: J Plankton Res. 2024 Sep 5;46(5):500–14. doi: 10.1093/plankt/fbae042 (PMC11443965; doi:10.1093/plankt/fbae042)
Supplement: Flo_et_al_JPR_Supporting_information_fdae042 [file flo_et_al_jpr_supporting_information_fdae042.docx]

# Supporting information

Title: Dietary plasticity in small Arctic copepods as revealed with brute force metabarcoding

Authors: Snorre Flo^1,2^, Camilla Svensen^2^, Kim Præbel^2,3^, Bodil Annikki Ulla Barbro Bluhm^2^, Anna Vader^1^

Supplementary Figures


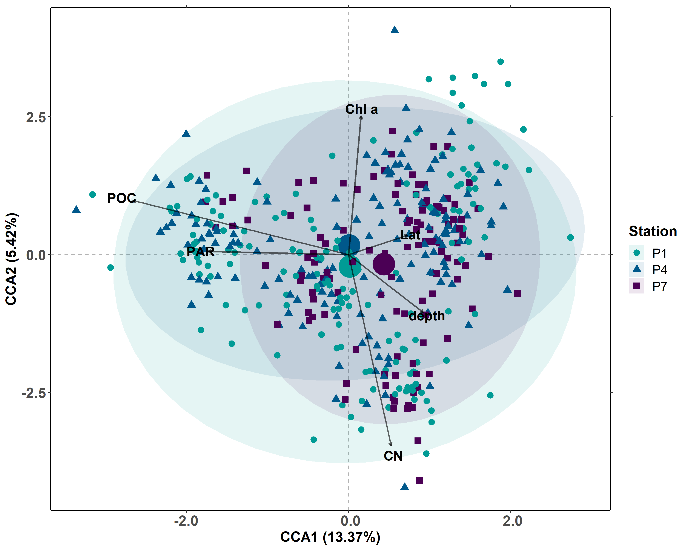

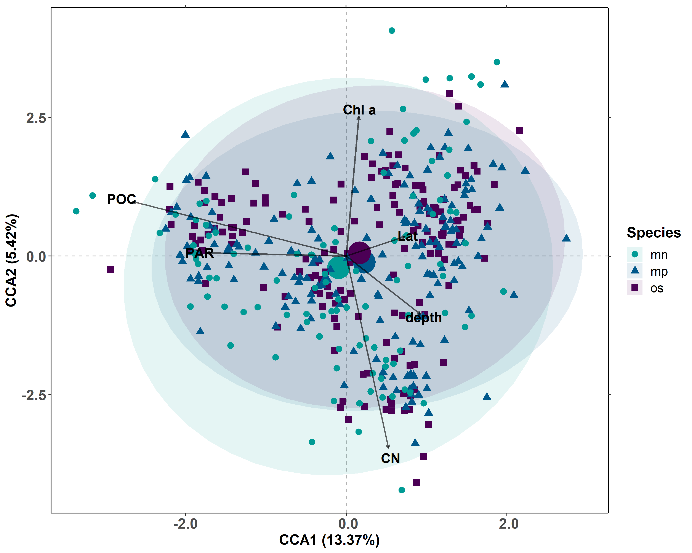


Fig. S1: CCA of all samples with a) stations and b) species and accompanying centroids of each group labelled by color.
